# Analog Sensor Interface for Field Mill Sensors in Atmospheric Applications

**DOI:** 10.3390/s22218405

**Published:** 2022-11-01

**Authors:** Zoi Agorastou, Thomas Noulis, Stylianos Siskos

**Affiliations:** Electronics Laboratory, Physics Department, Aristotle University of Thessaloniki, 54124 Thessaloniki, Greece

**Keywords:** electric field sensing, atmospheric applications, shutter-type field mill, electric field mill sensor interface, system-on-chip, noise optimized, low power consumption

## Abstract

An overview of the electric field mill sensor specifications in applications related to the measurement of the atmospheric electric field was conducted. The different design approaches of the field mill sensor interface are presented and analyzed, while the sensitivity-related parameters of a field mill are discussed. The design of a non-complex analog sensor interface that can be employed for the measurement of the electric field in both fair and foul weather conditions, such as thunderstorms, is implemented using discrete components for experimental validation and is optimized in an integrated version in terms of noise and power consumption. Advanced noise simulations are conducted in a 180 nm CMOS process (XH018 XFAB). The energy-autonomous operation of the sensor for extended periods of time is made feasible due to the low power consumption of the front-end circuitry (165 μW at 3 V) as well as the proposed intermittent style of operation of the motor. The total sensing system is low power, and its realization is simple and cost-effective, while also offering adequate sensitivity (45 mV/kV/m), making it comparable to the existing works.

## 1. Introduction

The development of electric field measurement systems for various scientific and engineering applications, as well as for research purposes, is widespread and was extensively studied both in the past and in the last few decades. The dramatically increasing prospects of HVDC line utilization for power transmission in modern power systems has brought about the need to assess the electric environment (i.e., the electric field, the ion current density, and the space charge density) under the transmission lines to establish safety guidelines. Consequently, the knowledge of these parameters at and immediately above ground level is of great significance due to the possible effects on health and safety during operation [1,2,3,4]. Electric field estimation is also important in dusty phenomena close to the earth surface [5], for aeronautical and astronautical projects and rocket launching [6], and for airborne measurements to create a profile of the fair weather electric field [7].

A major area of research associated with electric field measurement is the behavior of the electric field in meteorological and atmospheric phenomena with a special focus set on weather forecasting. In atmospheric electricity, most measured surface quantity is the vertical electric field, also known as the potential gradient [8]. The local electric field has been measured to provide a better understanding of thundercloud electrification and the energy exchange between lightning and the atmosphere across the globe [9]; to forecast lightning incidents; and to define an electric field threshold for lightning strikes on aircraft [10]. In [11], an investigation of electrical activity in thunderstorms was conducted, whereby the electrostatic field was measured during flights inside electrified clouds, in which lightning strikes occur, to provide insight beyond that of surface measurements. In fair weather conditions, the disturbance of the atmospheric electric field indicates the presence of aerosols [12], and strong variations of the field before earthquakes and during magnetic storms have been observed [13,14].

Early measurements of the near-surface, fair weather atmospheric electric field in the absence of electrified clouds is around 100 V/m, maintained by the global electric circuit (GEC) [15]; the direction of the electric field in fair weather conditions at the surface is negative. When electrified clouds are present, the field can increase from a few kV/m to tens of kV/m as it is significantly influenced by the cloud’s charge regions and lightning neutralization of the charges [9].

Various types of DC electric field sensors have been developed for science and engineering projects. In general, electric field meters provide either the magnitude of the electric field in V/m or the potential relative to a ground reference. The primary devices employed for electric field measurement are induction probes, field mills based on charge induction, and optical sensors.

The induction probe measures the potential of a conductive plate or antenna that has been equilibrated to the local ambient field with respect to a reference ground plane. However, the employment of induction probes for long-term electric field measurements is not preferrable, due to their susceptibility to ambient space charges and the need for frequent re-zeroing in shielded conditions [16]. As the challenging part of DC field measurement is the accumulation of random space charges near the sensors that interfere with the measurement of the electric field, for the construction of most sensors oscillating or vibrating devices are employed to create pseudo-AC fields. The conversion from a DC electric field to an AC signal benefits measurements by minimizing the effects of the space charge buildup and the spurious offsets. These devices operate either by alternately shielding or exposing a conductive plate, periodically exposing it to the local field, or by oscillating the top plate of a parallel-plate capacitor. They are advantageous compared to induction probes in that they provide higher sensitivity and they do not require constant re-zeroing.

The three prevalent sensors that adhere to this principle of operation are the shutter-type electric field mill, the vibrating plate electric field meter and the cylindrical field mill [5,17]. The electric field is determined by the measurement of the modulated capacitively induced charges or currents that are sensed by the conducting electrodes. The complexity in the manufacturing process of the existing electric field sensors, such as the cylindrical field mill, as well as their volume and the costly components, is not compatible with mass production. Additionally, although the presence of the space charge complicates electric field measurements in general, it is comparatively less difficult to measure electric fields at ground level than those at points above ground with cylindrical field mills. While optical sensors offer fast response and low noise, they also offer a limited dynamic range and require a complicated and costly manufacturing process [18,19,20].

Therefore, the instrument of choice for the electric field measurement in atmospheric applications is the electric field mill or shutter-type field mill, since its construction is non-complex; it offers a wide dynamic measurement range; it is characterized by stable sensitivity, good signal-to-noise-ratio, and fast response; and it is suitable for mass production.

Extensive research can be found regarding the improvement of various aspects of the field mill structure, its proper installation, and its effective operation monitoring. Nevertheless, limited works address the noise introduced by the sensor interface circuitry and especially the preamplification stage, while the challenges that arise due to the weak signal induced by the sensor and its low frequency (order of Hz) have not been adequately analyzed.

In this paper, an overview of the electric field mill sensor characteristics and its most important sensitivity-related parameters is provided. In addition, the most prevalent field mill sensor interface topologies are presented. A low-power design of the front-end circuitry for a field mill sensor is proposed. The main goal of the presented work was to create a sensor interface that satisfies the specifications defined in the atmospheric electric field measurement applications.

One of the requirements taken into consideration throughout the design process of the field mill sensor and its front-end circuitry is the capability of the sensor to measure electric fields with a range sufficient to include both near-surface fair weather and foul weather electric fields. Consequently, the sensor should provide adequate sensitivity and resolution to measure fields of several orders of magnitude efficiently and accurately. Furthermore, the energy-autonomous operation of the sensor for extended periods of time is essential since the installation is frequently in isolated environments where maintenance is difficult. Furthermore, a cost-effective sensor design should not significantly load the motor of the sensor, and the interface circuit design should be easily realized, utilizing commercially available discrete ICs, while simultaneously being easily integrated using standard IC fabrication processes. Additionally, the sensor and its interface should consume minimal power in order for it to be feasible to supply their operation through energy-harvesting systems. Integrating the sensor interface design allows the customization of the preamplification stage, while simultaneously offering a low-power and compact design solution. 

To address these requirements, the integrated version of the proposed sensor interface included a noise-optimized op-amp for the preamplification stage that sets the noise threshold low. For the sensor interface to be designed using discrete components while also providing the feasibility of integration, an alternative simplified approach to its design—i.e., the phase-sensitive detection stage—was adopted. An intermittent style of operation is assumed; this will prolong the energy-autonomous operation of the sensing system and contribute, along with the low-power circuitry, to the avoidance of the need for frequent battery replacement. The overall performance of the sensor interface is optimized to be comparable to the existing designs found in the literature or to outperform them in terms of resolution, sensitivity, and power consumption.

## 2. Electric Field Mill Sensor and Front-End Circuitry

### 2.1. Electric Field Mill Sensor

**Figure 1:**
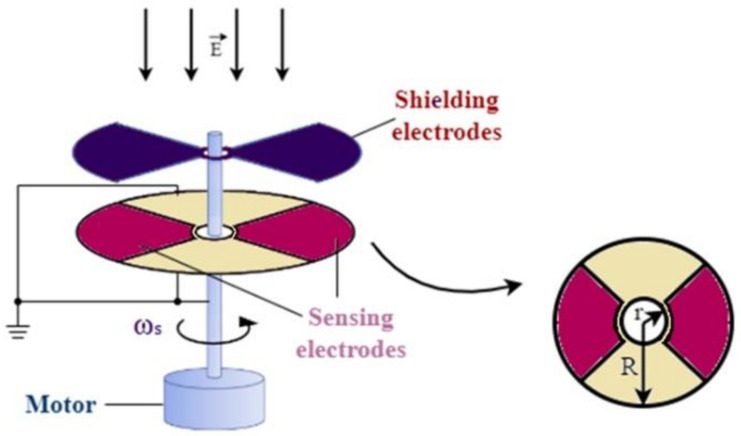
Shutter-type electric field mill sensor.

Figure 1 depicts the structure of the shutter-type electric field mill sensor. Harnwell and Van Voorhis developed one of the first known designs of an electric field mill [21]. This sensor, which is also known as the rotating electric field mill, or simply the electric field mill, is formed by two coaxially fixed and mutually insulated round plate-type electrodes; a sector-shaped shutter (also known as a rotor or chopper) consisting of the shielding electrodes, which is electrically grounded and rotates with the use of a motor with a constant speed; and a stationary segmented conducting plate—most commonly—consisting of sector-shaped sensing electrodes (occasionally referred to as stator). The rotating shutter periodically exposes and covers the sensing electrodes from the ambient electric field, and therefore, the charge is accumulated during the exposed state and conducted away during the shielded state of the latter.

The induced charge or current is converted to a voltage signal and further amplified and processed so that its amplitude, which is proportional to the ambient electric field, is extracted. The minimum electric field that can be measured is confined by the Johnson noise at the input resistor; the shot and fluctuation noise introduced at the preamplification stage; and the noise caused by the brushes employed to earth the rotating chopper and by long-term variation of the surface contact potentials surrounding the sensing region and by the pick-up of any signals harmonically related to the chopping frequency, such as the motor commutation noise and the optical encoder signals. AC coupling with low-frequency roll-off between the amplifier stages and phase-sensitive detection tackle the shot and fluctuation noise [22,23].

As this work focuses mainly on the optimization of the preamplifier in terms of the noise that limits its performance, specific field mill sensor parameters are provided below; these are correlated to the sensitivity of the sensor and/or the power consumption of the total sensing system.

#### 2.1.1. Gaps between Components

Adequate space between the sensing electrodes, the chopper, and the guard rings, is required. The separation gaps between the active surfaces in the field mill are necessary to minimize bridging (shorting) by precipitation, which leads to leakage currents that affect the measurements [7,23,24]. Fringing effects that create harmonic content can be caused by the fact that the shielding electrodes are on a different plane from the sensing electrodes, and they are enhanced with the decrease in the gap distance between them. Therefore, a reasonable distance between the chopper and the sensing electrodes is important to reduce the electric field generated by surface contact potential and to avoid zero-setting variations [22]. Another aspect that should be considered in determining the gap distance between the sensing electrodes and the shielding electrodes (rotor) is the trade-off between the gap and the induced charge. In [25], the experimental measurements showed that an increase in gap distance results in a decrease in the induced electric charge due to the edge effect, which depends on the gap distance.

Therefore, while a small distance gap leads to higher sensitivity, the environmental conditions as well as the challenges arising during assembly and operation when the proximity of the electrodes is significant, should be addressed.

#### 2.1.2. Sensor Sensitivity

An additional way to manage the noise produced by the contact potentials is to carefully select the materials of the rotor and the stator, which should not be easily contaminated by metal oxidation or surface dirt [26].

The magnitude of the electrostatic field is measured by measuring the induced charge, $q_{s}$, on the sensing electrodes:

(1) $$q_{s}=\int \bar{D}dA=\varepsilon_{0}ΕA$$

where $\bar{D}$ is the electric flux intensity, *A* is the area of the sensing electrodes, $\varepsilon_{0}$ is the absolute dielectric permittivity (8.85 × 10^−12^ Fm^−1^), and *E* is the electric field. Assuming a uniform electric field, the induced current is found by taking the derivative [27]:

(2) $$i_{s}=\frac{dq\left(t\right)}{dt}=\varepsilon_{0}Ε\frac{dA\left(t\right)}{dt}$$

The variation of the exposed electrode area depends on the shape of the stator electrodes. In the literature, most frequently found are the sector-shaped electrodes, which cause the area to vary linearly. Bernoulli lemniscate stators combined with a sector-shaped rotor are rarely used, whereas the combination of a stator made from circular plates and a rotor comprising circular holes appears in several projects [20]. The shape of the stator surface can result in harmonic content in the induced stator signal, i.e., odd harmonics of the signal frequency. These harmonics affect the raw current sensor signal and increase the signal-to-noise ratio [26].

For simplicity, the equations derived for the area variation, *A*(*t*), of the sector-shaped rotor and stator, which is the most frequently selected combination, are presented in [25]:

(3) $$A\left(t\right)=\left\{\begin{array}{c} \frac{1}{2}n\omega_{r}t\left(R^{2}-r^{2}\right)\;\;\;\;\;\;\;\;\;\;\;\;\;\;\;\;\;\;0\leq t\leq \frac{T}{2}\\ \left(\frac{\pi }{2}-\frac{1}{2}n\omega_{r}t\right)\left(R^{2}-r^{2}\right)\;\;\;\;\;\frac{T}{2}\leq t\leq T \end{array}\right.$$

The induced current, $i_{s}\left(t\right)$, can be derived from (2) and (3):

(4) $$i_{s}\left(t\right)=\left\{\begin{array}{c} \frac{1}{2}n\varepsilon_{0}\omega_{r}\left(R^{2}-r^{2}\right)Ε\;\;\;\;\;\;\;\;\;0\leq t\leq \frac{T}{2}\;\;\;\\ -\frac{1}{2}n\varepsilon_{0}\omega_{r}\left(R^{2}-r^{2}\right)Ε\;\;\;\;\;\;\frac{T}{2}\leq t\leq T \end{array}\right.$$

where $T=\frac{2\pi }{n\omega_{r}}$, ω*_r_* is the rotation speed, and *n* is the number of vanes of the sensing electrodes.

From (4), it is observed that the sensitivity of the sensor is dependent on the number of electrode vanes, on the angular frequency of the sensor, and the vane area. Therefore, theoretically, the sensitivity of a field mill sensor increases with the number of vanes, the surface area of the sensing electrodes, and the rotation speed. However, in [25], experimental measurements of induced current dependence on vane number showed that an increase in the number of openings results in an increased induced current, but only until the point where the edge effect prevails. The edge effect increases with the number of vanes and causes a decrease in the electric field intensity. Field mills consisting of two to eight vanes are more frequently employed [3,9,18,20,28,29,30,31]. 

While providing a higher sensitivity, a large surface area of the sensing electrodes would consequentially require the rotor vanes to have a large area, which would result in a bulky sensor design. Taking into consideration that a bulky rotor design would result in higher loading of the motor and therefore higher power consumption of the motor, smaller electrode dimensions are preferred in self-powered field mill sensors. The diameters of the electrode plate vary from tens to hundreds of millimeters (30 mm [25,28], 85 mm [3], 114 mm [7], 115 mm [9]).

#### 2.1.3. Rotating Frequency

While selecting a motor that would provide a high chopping frequency would result in low noise signals, higher motor power consumption would be required, and the earthing brush wear would increase [22]. In [9], the rotational speed of 33.3 Hz was selected to balance time resolution, power consumption, and mechanical longevity, while in [28] the rotational speed of 60 Hz was achieved by regulating the motor supply at 3.3 V for the minimization of the current consumption and was adequate to provide a measurable current in a fair weather electric field measurement application. Mostly rotational speed varies from a few tens of Hz (50 Hz [25]) to a few hundreds of Hz since most low-cost motors rotate at that rate.

The field mill sensor employed for this work was designed with a large sensing electrode area to provide high sensitivity, and it operated at a low rotation speed to mitigate the motor power consumption.

### 2.2. Conventional Electric Field Mill Sensor Interface

**Figure 2:**
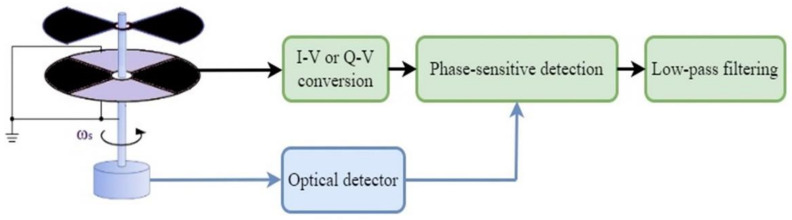
Typical field mill sensor interface—single-ended measurement.

A typical field mill sensor interface sequence of circuits is depicted in Figure 2.

A detailed description of each stage is provided below.

#### 2.2.1. Preamplification Stage

The typical sensor interface of an electric field mill consists of a preamplifier which converts the induced charge or current into a voltage and provides amplification of the weak current signal output of the electrodes. A charge [7,10,25,32,33] or a transimpedance [3,9] amplifier is used in most of the works; it should introduce minimum noise and have low input bias currents since the induced current is typically comparable in value to the leakage currents, and the field measurement would be significantly affected. An additional voltage gain stage is incorporated in several interfaces [7,28].

In some works, the RC network of the charge amplifier is selected so that the output voltage signal is independent of the frequency of the sensor rotation [10,33], which is a desirable feature since the motor rotation speed might vary slightly during operation.

The op-amp of the preamplification stage should be carefully selected or designed since it essentially establishes the sensitivity of the entire sensor interface. 

#### 2.2.2. Phase-Sensitive Detection—Synchronous Demodulation

A synchronous detector (phase-sensitive detector) is employed to synchronously demodulate the signal. An optical switch, which senses the motor rotation, is included in most cases to provide a reference signal for the phase-locked signal detection [3,20]. This signal indicates a specific angular motor position or the state of exposure of the sensing electrodes, as well as the rotation speed of the motor. The phase-sensitive detection is employed to extract both the amplitude and the polarity of the ambient electric field and can be realized by a lock-in amplifier [34]. An extra benefit of phase-sensitive detection is the reduction in noise since at this stage only the frequency of the induced signal is amplified and, in some applications, a bandpass filter is also employed to complement the filtering of noise. This phase-sensitive detection stage is implemented either in the analog or in the digital domain and several variations of it exist in the literature. As mentioned in [26], the employment of synchronous detection does not mitigate the noise caused by the existence of the odd harmonics since these frequencies are multiplied by a square which contains the same odd harmonics (since the square signal is the reference signal extracted by the optical encoder and has the frequency of the induced current signal).

An analog op-amp demodulator with a ± 1 gain is used in [24]; it is phase-locked to the rotation of the shutter. In [9], the signal generated by the optical encoder is sampled and recorded by an ADC to increase flexibility at post-processing and decrease the power consumption of the analog circuitry. Both the optical encoder signal and the sensor signal are filtered in the same manner in order to end up with their original phase difference, which indicates the field polarity. In [3], the phase-sensitive detection circuit is described as a two-way switch controlled by the reference signal, which is, essentially, mathematically equivalent to the product of the sensor signal and the optical encoder signal. For the latter, a phase-shifting circuit is employed to maximize the sensitivity. In this work, the logic used in the configurations above is combined and expanded to create an alternative phase-sensitive detection method.

An alternative method to extract the polarity of the electric field that excludes the photoelectric detector is presented in [35], where the commutating pulse signal of the DC motor is acquired synchronously with the electric field signal to detect the phase of the rotor.

#### 2.2.3. Low-Pass Filtering

After the phase-sensitive detection, the rectified signal is typically low-pass filtered and, in some cases, fed to an analog-to-digital converter (ADC). A microcontroller can be employed for the organization of the data and their serial transmission, as well as to record various parameters for maintenance purposes [7], to achieve constant rotation speed for the motor [3], and to activate/deactivate the motor and select the post-demodulation gain [28].

#### 2.2.4. Power Supply

The power supply for the analog circuitry in most applications is implemented by bipolar power rails [9,25,28] since the demodulation stage of the signal requires either the use of a ± 1 gain of amplification or the mathematical equivalent of multiplication, where the different polarity of the electric field is expressed by positive and negative output voltages. However, the use of a symmetrical power supply is limited in modern applications and is not compatible with the current trends in single-supply operation.

### 2.3. Calibration Techniques

**Figure 3:**
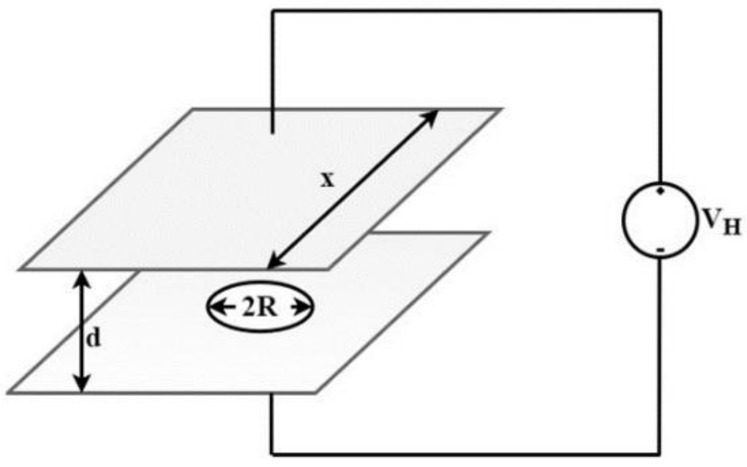
Calibration setup.

The calibration setup of the field mill sensor is conducted by exposing the device to a known electric field, which is created by two parallel conducting plates and a variable high-voltage power supply that applies a high voltage across the plates (Figure 3) [7]. To avoid distortion of the known uniform field, a hole in one of the plates is opened and the sensor is mounted flush with it. The IEEE guidelines for the appropriate dimensions of the conducting plates and the correct distance between them, which are relative to the sensor dimensions, can be found in [36]; to avoid perturbation of the surface charge density on the top plate, the distance, *d*, between the plates should be at least three times the radius, *R*, of the field mill sensor.

The resulting electric field mill is:

(5) $$E=\frac{V_{H}}{d}$$

In [35], a self-calibration technique is realized using an additional electrode with a fixed distance from the sensing electrode and applying a known voltage to it to create an electric field for absolute calibration.

## 3. Proposed Electric Field Mill Sensor and Front-End Circuitry

### 3.1. Field Mill Design

**Figure 4:**
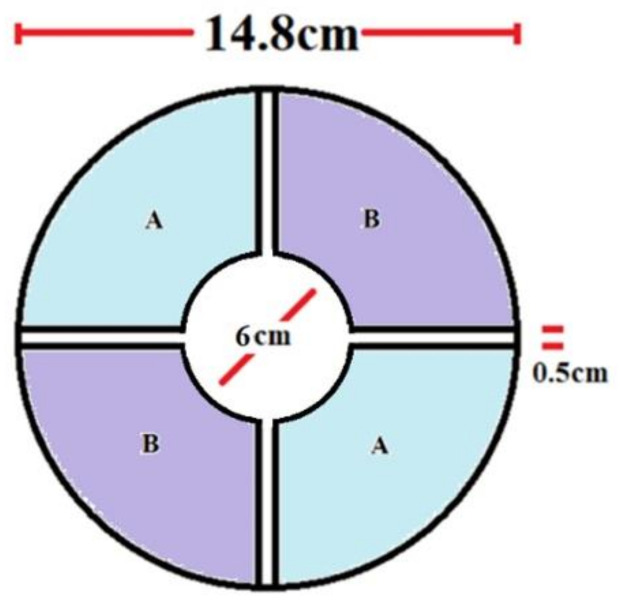
Field mill sensor top view.

The top view of the electric field mill sensor sensing electrodes, whose specifications determined the design of the front-end stage of the sensor interface, is depicted in Figure 4. Two sets of sensing electrodes, set A and set B, each of which consists of two vanes, are utilized for the differential measurement. The current signal induced by set A will have a phase difference of 180° from the current induced by set B.

The main parameters of the field mill sensor appear in Table 1.

The dynamic range of the measurement should be adequate to cover both fair-feather electric fields and fields during thunderstorms while providing high accuracy. Therefore, a field mill that can measure accurately ±20 kV/m with a resolution of 10 V/m would be sufficient.

Using the above parameters for the theoretically expected induced current for an electric field mill results in currents of the order of 320 pA for fair weather conditions, where the electric field is in the hundreds of V/m, and of approximately 64.8 nA for foul weather conditions. Therefore, a low voltage and current noise density of the op-amp used for the preamplifier realization is essential. 

The application under study employs a motor that operates intermittently, i.e., is idle (standby mode) for most of the time and is only activated each time for a few seconds. The average current consumption of this style of operation is calculated by:

(6) $$I_{avg}=\frac{I_{OPE}\times T_{OPE}+I_{IDLE}\times T_{IDLE}}{T_{OPE}\times T_{IDLE}}$$

Therefore, for a duration of operation of 5 s and a 55-s idle state, which is an adequate duration both for acquiring a measurement and for detecting changes of the electric field before thunderstorms, the motor would consume approximately 0.65 mA. As a result, the contribution of the sensor interface circuits becomes significant in the total power consumption and should be minimized to increase the life of the energy-autonomous operation of the sensing device.

The proposed sensor interface includes an alternative simplified version of the phase-sensitive detection circuit, instead of the analog multiplier, the synchronous demodulator, or the lock-in amplifier commonly used in most works. The interface can be optionally combined with a nano-power microcontroller unit (MCU) (e.g., MSP430 series by Texas Instruments), which can control the motor activation and contribute to signal processing.

### 3.2. Front-End Circuits

**Figure 5:**
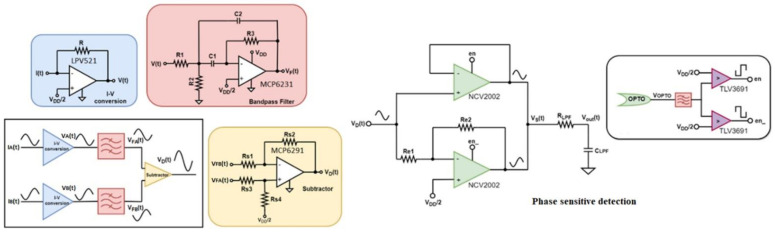
Design of the sensor interface.

Figure 5 depicts the proposed sensor interface, which consists of a preamplification stage, a filtering stage, and a phase-sensitive detection stage. An optical encoder, which generates a signal, *V_OPTO_*, that indicates the exposure state of the sensing electrodes, is also employed for the electric field sign extraction.

The preamplification stage is realized by two separate channels, one for each set of sensing electrodes, each of which consists of a transimpedance amplifier that converts the current signal induced by each set into a voltage signal. The relation for the gain the transimpedance amplifier provides is:

(7) $$V_{X}=RI_{X}$$

where *R* is the feedback resistance, and the subscript *X* can represent either electrode set A or electrode set B. It should be noted that the transimpedance amplifier does not cause a phase shift to the original sensor signal, which translates to a 0° phase difference between the output voltage signal, *V_A_*(*t*), and the current of electrode set A, *I_A_*(*t*), and the corresponding signals of channel B.

Bandpass filtering of the signal was considered necessary since the induced signal from the electrodes, which has an amplitude proportional to the field magnitude, is expected to have a frequency equal to the rotation frequency multiplied by the number of vanes:

(8) $$f_{s}=nf_{r}$$

A multiple feedback narrow bandpass filter for each channel, whose central frequency was around the expected frequency of the signal, which is 25.5 Hz according to (10), was selected to exclude unwanted spurious signals as well as slow linear trends in the data. An additional benefit of the narrow bandpass filter is the exclusion of the odd harmonics of the signal frequency, which are caused by the shape of the stator. All frequencies, except for the frequency derived by (10), are filtered out.

The filtering stage contributes to the amplification of the voltage signal. Additionally, the filter causes a phase shift to the original sensor signal.

A voltage subtractor provides the difference, *V_D_*(*t*), of the signals *V_FA_*(*t*) and *V_FB_*(*t*).

The final stage of the sensor interface is the phase-sensitive detection, which, combined with the processed optical encoder signal, provides an output voltage signal that contains information on both the magnitude and the polarity of the electric field. This configuration uses the same logic as in [9], where the optical encoder signal is processed in the digital domain in the same manner as the raw sensor signal and used as a reference to extract the field polarity by its phase difference. In this work, the realization of the optical encoder signal processing is conducted in the analog domain.

From Figure 5, it can be observed that the phase-sensitive detection stage consists of two operational amplifiers with a shutdown feature, i.e., a high signal activates the op-amp operation, while a low signal deactivates it. The first op-amp is in inverse amplifier configuration, while the second is in a unit-gain (buffer) configuration.

As neither the transimpedance amplifier nor the voltage subtractor cause a delay to the raw sensor signal and the only phase shift is introduced by the bandpass filter, filtering the signal generated by the optical sensor in the same manner as the raw sensor signal results in an approximately 0° phase difference between the processed optical encoder signal and the filtered sensor signal for the positive electric fields and an approximately 180° phase difference for the negative electric fields.

The filtered optical encoder signal is passed through a comparator that generates a clean square pulse waveform, en, that controls the activation of the buffer amplifier and its inverted version, en, which is responsible for the activation of the inverting amplifier. When the optical encoder signal is high, the unity gain amplifier is activated by en, and the inverting amplifier is deactivated by en_; therefore, for the respected duration the output signal is the same as the input signal, *VD(t)*. When *VOPTO* is low, it activates the inverting amplifier, and the output signal is the inverted input signal for the respected duration. A low-pass filter is employed to convert the derived voltage signal, *V_S_(t)*, into a DC voltage, *V_out_*, which is proportional to the ambient electric field. Observing the phase-sensitive detection configuration, the derived conclusion is that voltage *V_out_* will be higher than the mid-supply level (VDD/2) when the electric field is positive, and vice versa.

## 4. Experimental Measurements

The experimental measurements to verify the operation of the proposed field mill interface depicted in Figure 5 were conducted on a PCB containing a discrete implementation of the proposed sensor interface using a prototype 3D-printed electric field mill sensor. The motor had a rotation frequency of 12.75 Hz when operating at 5 V. A calibration setup with similar structure to the one depicted in Figure 3 was employed to create known electric fields ranging from −6.5 kV/m to +6.5 kV/m.

Table 2 includes the values of all the passive components used.

Discrete ICs that are characterized by low noise and low power consumption were used for the preamplification stage, the bandpass filter, and the phase-sensitive detection stage, whose models appear in Figure 5.

**Figure 6:**
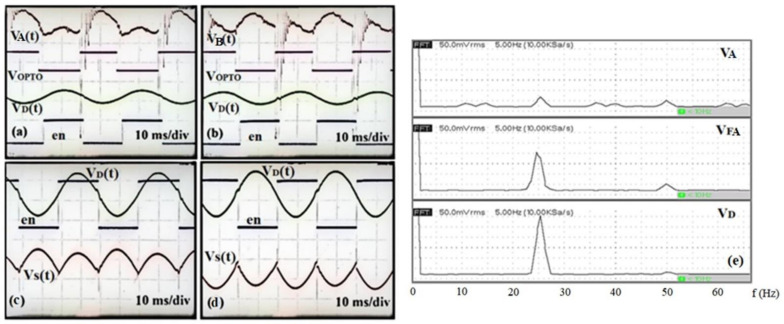
(**a**–**d**) Oscilloscope measurements; (**e**) amplitude-frequency response characteristics analysis (Hanning window function).

The oscilloscope measurements appear in Figure 6a–d, where the top two measurement windows in Figure 6a,b show the sensor signal and the optical encoder signal before (*V*_1_(*t*) and *V_OPTO_*) and after processing (*V_D_*(*t*) and en) for the positive and negative electric fields, respectively. The bottom two measurement windows in Figure 6c,d show the differential signal after subtraction, *V_D_*(*t*), along with the processed optical encoder signal, en, and the output waveform of the phase-sensitive detection circuit, *V_S_*(*t*), before low-pass filtering. To analyze the amplitude-frequency response, an FFT of the voltage waveform at each processing stage is performed using a digital storage oscilloscope (SIGLENT, *SDS1102CML+*). The derived diagrams are depicted in Figure 6e.

As can be observed in Figure 6e, before filtering the voltage signal *V_A_*(*t*) contains several unwanted frequency components caused by both environmental (external) noise sources and internal circuitry noise contribution. After filtering, the voltage signal *V_FA_(t)* contains only the desired frequency of 25.5 Hz amplified by the bandpass filter and a 50 Hz common-mode noise picked up by both electrodes. After the voltage subtraction, the remaining frequency is the theoretically expected frequency of 25.5 Hz, and the signal is doubled in amplitude. The 50 Hz common-mode noise is greatly eliminated.

**Figure 7:**
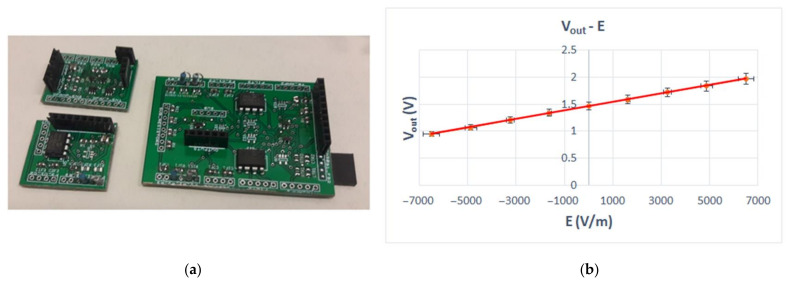
(**a**) Discrete implementation of the proposed sensor interface; (**b**) output voltage vs. electric field (linear equation: *V_out_ =* 8 × 10^−5^ *E +* 1.4622 V, *R^2^ =* 0.9998).

Figure 7a depicts the discrete implementation of the proposed sensor interface and the calibration setup. In the diagram of Figure 7b, the output voltage is plotted against the applied electric field.

## 5. Optimized IC Sensor Interface

**Figure 8:**
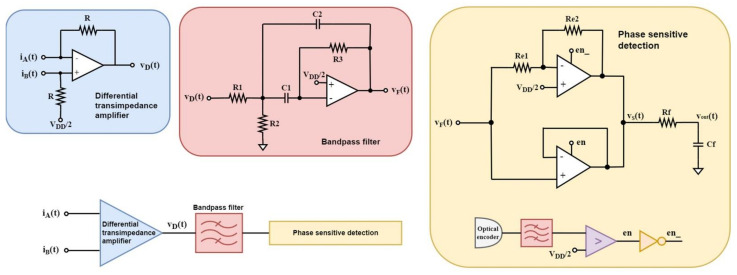
Proposed sensor interface.

The optimized integrated sensor interface appears in Figure 8. During simulations the theoretical model’s Equation (4) were used to simulate the current induced by the sensing electrodes. 

The values for each passive component of Figure 8 appear in Table 3.

The implementation of the integrated version of the sensor interface has some slight design differences from the discrete version, in order to improve the silicon area consumption. An alternative differential transimpedance amplifier configuration [37] was selected for the integrated implementation instead of the classic configuration that consists of one separate amplification channel for each set of electrodes since it provides differential-to-single-ended signal amplification, employing only one operational amplifier and, therefore, decreasing the power consumption, the silicon real estate, and the need for an op-amp subtractor, as well as the need for a second bandpass filter, is avoided. The relation for the gain the transimpedance amplifier provides, is:

(9) $$V_{D}=2R\left(i_{A}-i_{B}\right)$$

**Figure 9:**
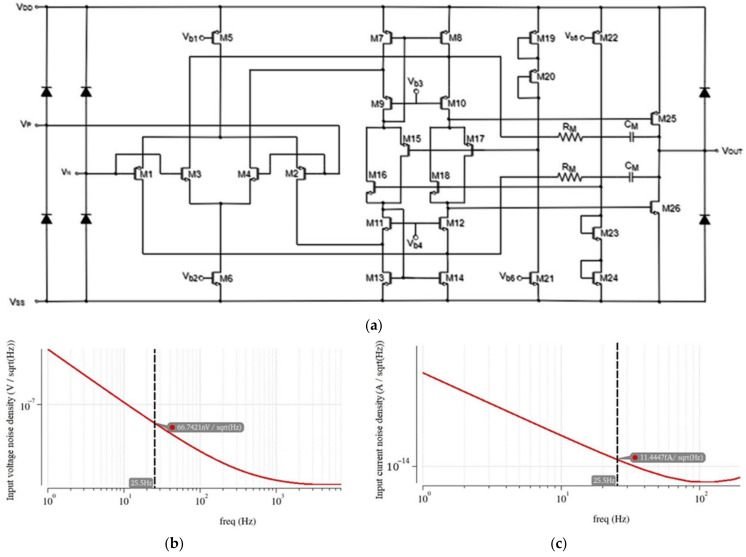
(**a**) Schematic of op-amp used for the preamplification stage; (**b**) input voltage noise density; (**c**) input current noise density.

Low noise introduction in the preamplification stage is of great significance in applications where accurate measurements are necessary since the minimum electric field that can be measured depends on this noise. Therefore, a custom-designed operational amplifier optimized for the specific requirements of low noise combined with low power consumption was employed for this stage (Figure 9a) [38]. As a low-voltage operation combined with low power consumption was required for the integrated version of the proposed sensor interface, a compact amplifier that combines low voltage and power efficiency was needed. This amplifier’s output rail-to-rail configuration is necessary to maximize the dynamic range and, by extension, the signal-to-noise ratio. The floating class AB driver prevents the contribution of the class AB stage to the noise and offset introduced by the amplifier [39].

The dimensions of the optimized op-amp MOSFETs appear in Table 4. The Miller compensation capacitor C_M_ is 12 pF, and the Miller compensation resistor is 1.5 kΩ.

Advanced noise simulations were conducted (Figure 9b,c), and the final design parameters appear in Table 4. The signal induced from the sensing electrodes has a low frequency (in the order of tens of Hz). At this frequency band, a flicker noise is the dominant source of noise, and therefore, input transistors with a large *WL* area are required to reduce its contribution. A trade-off between the current consumption and the noise introduced by the differential pairs exists, where an increased current results in decreased noise [40]. Low power is also a requirement; hence, the current at the differential pair of the op-amp was selected as a good balance between the consumption and the input voltage noise.

The fabrication technology transistor models (XFAB 0.18 μm) that were employed for the design of the amplifier are supplied in the BSIM3V3 format. BSIM3V3 contains advanced thermal and flicker noise models [41].

To calculate the noise output in closed-loop op-amps, the noisy op-amp is modeled as a noiseless op-amp with an equivalent input-referred, mean-square noise voltage. The effect of all the noise sources in the circuit is represented by a single source at the input.

A hands-on simulation-based methodology for the noise optimization of the amplifier utilizing the advanced noise simulation tools provided by Cadence was conducted. Noise simulators provide the possibility to determine not only the equivalent input-referred noise, but also the type of noise sources that constitute the extracted noise and the noise contribution of each component. Using these features combined with the theory on noise sources, the efficient noise optimization of the amplifier was feasible. 

At first, during the initial advanced noise analyses of the operational amplifier before the optimization of the design, it was confirmed that the prevailing noise sources were the flicker noise and the thermal noise. This observation is consistent with the operational amplifier noise theory. In addition, according to [39], the noise of the amplifier is determined mainly by the input transistors and the summing circuit since the floating class-AB control is shifted into the summing circuit. This was confirmed by simulations of the noise contribution of each component.

The dominant noise source in the frequency domain of the induced sensor signal—which is in the order of tens of Hz—is the flicker (or 1/*f*) noise. Flicker noise is described by the following equation:

(10) $$V_{g}^{2}\left(f\right)=\frac{K}{WLC_{ox}f}$$

where *K* is a process dependent constant, *W* and *L* are the transistor’s width and length, respectively, and *Cox* is its gate capacitance per unit area. This was confirmed by noise simulations of the amplifier, where in low frequencies the noise decreases at a rate of 10 dB/decade, which is in accordance with the flicker noise behavior. Thermal noise is caused by the random thermally excited vibration of the charge carrier in a conductor. It is present in all resistive elements, and it is dependent on the absolute temperature. A the MOSFET level, it is described by the following equation:

(11) $$I_{d}^{2}\left(f\right)=\left\{\begin{array}{c} \frac{4kT}{r_{ds}},\;\;\;\;\;\;\;\;\;\;\;\;\;\;\;\;\;\;\;\;triode\;region\\ 4kT\gamma g_{m},\;\;\;\;\;\;\;\;\;\;\;\;\;active\;region \end{array}\right.$$

where k is the Boltzmann’s constant, *T* is the temperature in Kelvin, *r_ds_* is the channel resistance, and *g_m_* is the device transconductance [42]. The input-referred voltage noise density at 25.5 Hz at this stage was 1.25 μV/√Hz.

In the next step, the noise contribution of each transistor at the frequency of interest (25.5 Hz) was extracted to determine the prevailing noise contributors. The differential pair transistors presented the highest noise contribution percentage; therefore, according to the flicker noise equation, increasing their *WL* area, would decrease their noise contribution. For that reason, parametric simulations sweeping the *WL* area of the input transistors were conducted to determine the dimensions of each transistor finger. The voltage noise density at 25.5 Hz at this stage was 363.2 nV/√Hz.

To further decrease the noise contributions of the input stage, the width of the transistors was increased. Parametric simulations sweeping the number of fingers (*n_f*) of the input transistors were conducted to determine the total width of each transistor. The voltage noise density at 25.5 Hz at this stage was 233.3 nV/√Hz.

At this point in the simulations, the noise contribution of the input transistors became comparable to the noise contribution of the transistors of the summing stage of the amplifier; therefore, a similar approach was adopted to decrease the noise introduced by this stage. Parametric simulations sweeping the area of transistors M7-M10 and M11-M14 were conducted. The voltage noise density at 25.5 Hz at this stage was 66.7 nV/√Hz.

**Figure 10:**
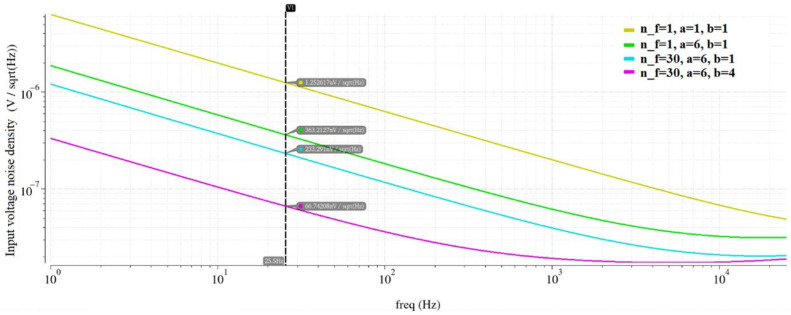
Noise optimization steps.

At this point, no further optimization of the design in terms of noise was conducted since the noise was adequately low. The final transistor dimensions appear in Table 4. Figure 10 sums up all the optimization stages conducted, where *n_f* is the number of fingers of the input transistors, a is the constant that is multiplied by both the nominal width and the nominal length of the input transistors, and b is the constant that is multiplied by both the nominal width and the nominal length of the summing circuit transistors M7-M10 and M11-M14.

The nominal dimensions for transistors M1–M2 and M7–M10 were *W =* 30 μm and *L* = 2 μm, and for M3–M4 and M11–M14, they were *W* = 10 μm and *L* = 2 μm.

Table 5 includes the noise parameters as well as the power consumption of the sensor interface.

**Figure 11:**
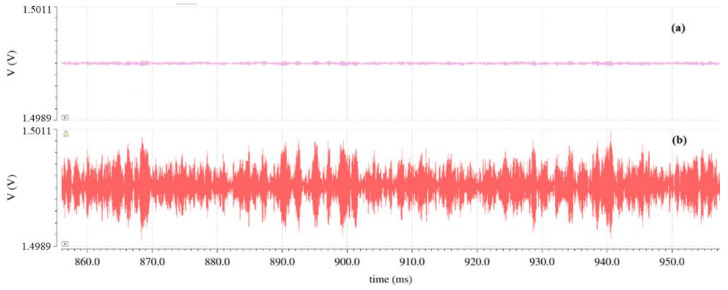
Transient noise analysis (**a**) at the preamplifier input and (**b**) at the preamplifier output.

To interpret the noise introduced by the preamplification stage, the transient noise of the preamplification stage is provided for the input and output node. In Figure 11, the transient noise analysis provides the peak-to-peak voltage noise.

The related amplitude, which is about 140 μV_p-p_ at the preamplifier’s input and approximately 1.6 mV_p-p_ at its output, is significant and concerns the preamplifier after optimization, depicting that the noise optimization of the preamplification stage is crucial.

**Figure 12:**
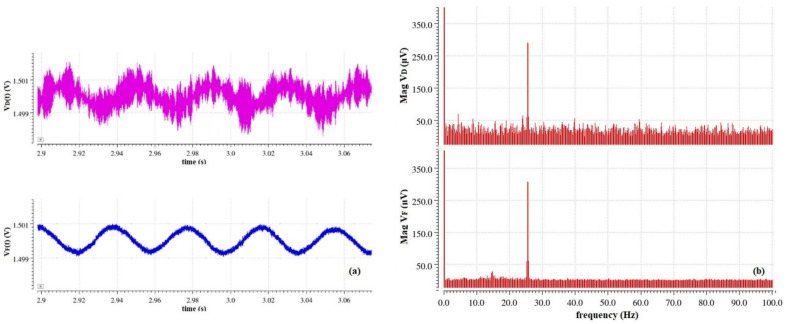
(**a**) Transient noise simulation of signal after I-V conversion and after filtering for *E* = 10 V/m; (**b**) FFT transform (Hanning window function).

Simulations including the transient noise set by the internal noise of the preamplification stage combined with the filtering stage were conducted—assuming an ideal noiseless environment—and a spectrum analysis to deconstruct the time domain representation of the derived signals into the frequency domain representation was performed (Figure 12). The spectrum analysis after the filtering stage showed significant elimination of the internal noise that is introduced by the preamplification stage.

The total power consumption of the sensor interface contributes significantly to the consumption of the total field mill sensor system due to the intermittent style of operation of the motor; therefore, to prolong its energy-autonomous operation, the sensing interface should consume as little as possible. From Table 4, it is derived that the preamplifier is the main contributor to the total power consumption, and therefore, its consumption should be limited.

The integration of the on-chip bandpass filter is a challenging process since dealing with low frequencies generally results in the filters requiring passive components with high values that are difficult to integrate. By selecting the minimum possible values for the capacitors (100 pF) with a high area capacitance (MIM capacitor) included in the filter configuration and high values for the resistors, as well as highly resistive components provided by the fabrication technology (high ohmic N+ poly resistor), the integration challenge is overcome. Low power 3.3 V nMOS and pMOS transistors were used throughout the whole interface design. 

**Figure 13:**
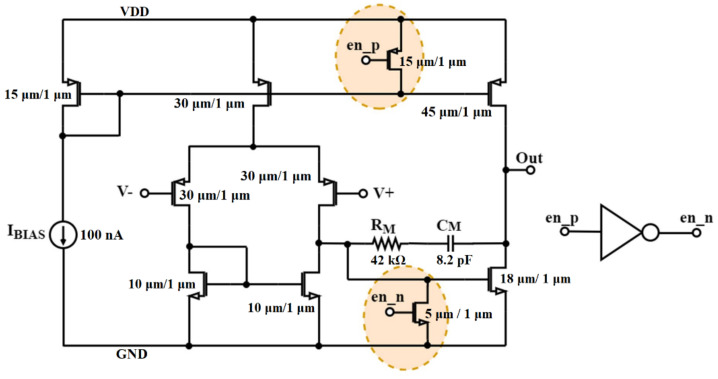
Schematic of op-amp with enable feature.

Figure 13 depicts the schematic of the op-amp circuit with the enable feature, where the control signals en_p and en_n are externally controlled by signals en and en_ for the buffer and the inverting amplifier configuration, respectively. The same op-amp design was employed for the implementation of the bandpass filter op-amp as well as the comparator, but without the enable feature.

**Figure 14:**
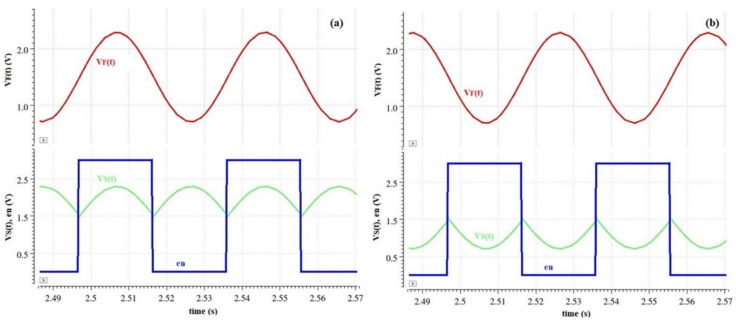
Phase-sensitive detection simulation signals for (**a**) positive and (**b**) negative electric field.

The main waveforms of the phase-sensitive detection stage appear in Figure 14.

**Figure 15:**
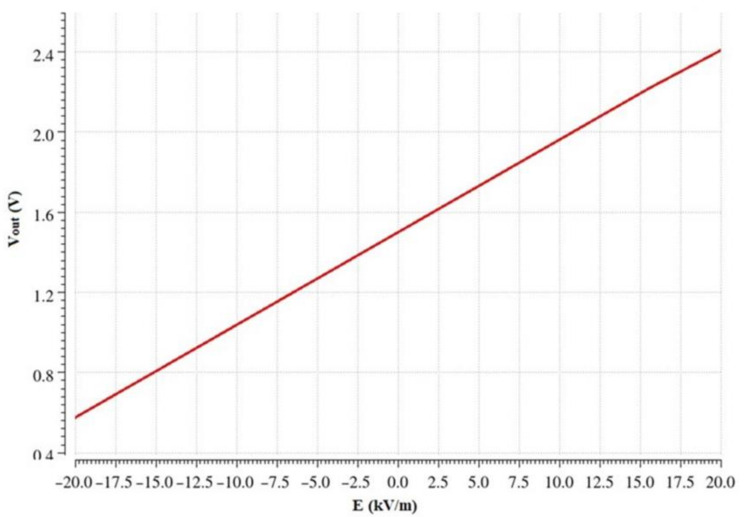
Output voltage vs. electric field (linear equation: *V_out_ =* 4 × 10^−5^ *E +* 1.4991 V, *R^2^* = 1).

The output voltage after low-pass filtering is plotted versus the electric field in Figure 15. The maximum achieved sensitivity is 45 mV/kV/m.

The main characteristics and performance parameters of the proposed sensor interface are compared in Table 6 with the other works in the literature that employ an electric field mill. 

From Table 6, it is apparent that the proposed sensor and its sensing interface consume significantly less power than the field mill sensing systems that are employed in foul weather measurements, even when they operate in continuous mode, whereas they provide high sensitivity combined with a single power supply of 3 V. The low power consumption aspect is crucial when an extended energy-autonomous operation is required. It should be noted that while an ADC was not employed in the design of the processing channel, the theoretical resolution of the interface, should a 16-bit ADC be used, was calculated and included in Table 6 for comparison reasons.

Using a relatively larger sensing electrode area combined with the low noise preamplification stage, an improved sensitivity and resolution are achieved, and accurate fair weather measurements become feasible even at a low rotation frequency of the motor. Therefore, a carefully designed field mill sensor that follows the guidelines discussed, combined with the proposed sensor interface would be ideal for measurements of both fair and foul weather conditions.

## 6. Conclusions

In this work, an overview on the electric field mill sensor’s sensitivity-related parameters, as well as the parameters that affect its power consumption, was conducted. The design parameters that should be taken into consideration during the design process of a sensor interface for an electric field mill in atmospheric applications were presented. A sensor interface that satisfied the requirements of this specific application was designed, experimentally tested, and optimized in an integrated version that provided improved performance in terms of noise and power consumption. For the realization of the front-end circuitry, an alternative phase-sensitive detection configuration combined with a narrow bandpass filter was designed, and FFT spectrum analyses were performed to demonstrate its contribution to both external and internal noise elimination. The achieved sensitivity of the sensor is 45 mV/kV/m, and the total power consumption of the interface circuitry is 165 μW.

## Figures and Tables

**Table 1:** Parameters of the electric field mill sensor used for measurements.

Parameter	Value	Unit
Number of vanes, *n*	2	-
Big radius, *R*	74	mm
Small radius, *r*	30	mm
Rotation frequency, *f*	12.75	Hz
Motor type	Brushless DC	-
Motor current consumption @ 5 V	36	mA

**Table 2:** Passive component values of the discrete implementation.

Component	Value	Unit
*R*	2	MΩ
*R*1	1	kΩ
*R*2	47	Ω
*R*3	10	kΩ
*C*1, *C*2	10	nF
*Rs*1, *Rs*2, *Rs*3, *Rs*4	100	kΩ
*Re*1, *Re*2	100	kΩ
*Rf* (external)	2.5	MΩ
*Cf* (external)	60	nF

**Table 3:** Passive component characteristics of the integrated implementation.

Component	Value	Unit	Dimensions on Chip
*R*	10	MΩ	*W* = 0.5 μm, *L* = 714 μm
*R*1	80	MΩ	*W* = 0.42 μm, *L* = 4.69 mm
*R*2	40	MΩ	*W* = 0.42 μm, *L* = 2.34 mm
*R*3	374	MΩ	5 × (*W* = 0.42 μm, *L* = 4.39 mm)
*C*1, *C*2	100.6	pF	49 × (*W* = 29.5 μm, *L* = 29.5 μm)
*Re*1, *Re*2	2.5	MΩ	*W* = 2 μm, *L* = 773 μm
*Rf* (external)	2.5	MΩ	-
*Cf* (external)	60	nF	-

**Table 4:** Preamplification op-amp MOSFET dimensions.

Transistor	Dimensions	Transistor	Dimensions
M1, M2	5400 μm/12 μm	M16	5 μm/1 μm
M3, M4	1800 μm/12 μm	M17	5 μm/1 μm
M5	18 μm/1 μm	M18	1.6 μm/1 μm
M6	6 μm/1 μm	M19, M20	30 μm/1 μm
M7-M10	120 μm/8 μm	M23, M24	10 μm/1 μm
M11-M14	40 μm/8 μm	M25	120 μm/2 μm
M15	15 μm/1 μm	M26	30 μm/2 μm

**Table 5:** Noise parameters and power consumption of the sensor interface.

Parameter	Value	Unit
Input voltage noise @ 25.5 Hz	66.7	nV/√Hz
Input current noise @ 25.5 Hz	11.4	fA/√Hz
Power consumption of preamplifier @ 3 V	124.6	μW
Total power consumption @ 3 V	165.6	μW

**Table 6:** Table of comparison of electric field mill sensor interface circuits in the literature.

Parameter	[43]	[9]	[28]	[10]	[20]	This Work
Number of sector-shaped vanes, *n*	2	3	2	6	Two circle-shaped sensing electrodes	2
Sensor dimensions	N/A	11.5 cm diameter	3 cm diameter	N/A	5 cm diameter of each electrode	15.2 cm diameter
Electric field range	N/A	N/A	±150 V/m	0–80 kV/m	1.5 kV/m (high-sensitivity channel) 15 kV/m (low-sensitivity channel)	±20 kV/m
Sensitivity	N/A	N/A	~1 mV/V/m	48.75 mV/kV/m	N/A	45 mV/kV/m
Resolution	16 bits	16 bits 2 V/m	16 bits	N/A	3 V/m (high-sensitivity channel) 30 V/m (low-sensitivity channel)	16 bits 0.6 V/m
Rotation frequency, *f* (Hz)	5	33.3	60	N/A	N/A	12.75
Motor type	Low noise, brushless	Three-phase motor Maxxon EC32	N/A	Brushless DC motor	Step motor	Brushless DC motor
Power supply	12 V	8 V motor and motor driver 5 V digital systems and sensors ±5 V analog circuitry	6 V	>4 V	N/A	3 V analog front-end supply 5 V motor supply
Power consumption	1.6 W	4 W	408 mW (264 mW motor consumption)	N/A	1.8 W	Continuous operation: 180.165 mW Intermittent operation: 3.435 mW

## Data Availability

Not applicable.
